# Perfectly Active Teenagers. When Does Physical Exercise Help Psychological Well-Being in Adolescents?

**DOI:** 10.3390/ijerph16224525

**Published:** 2019-11-15

**Authors:** Juan González-Hernández, Manuel Gómez-López, José Antonio Pérez-Turpin, Antonio Jesús Muñoz-Villena, Eliseo Andreu-Cabrera

**Affiliations:** 1 Department of Personality, Evaluation and Psychological Treatment, University of Granada, 18071 Granada, Spain; jgonzalez@ugr.es; 2 Department of Physical Activity and Sports, University of Murcia, Santiago de la Ribera, 30720 Murcia, Spain; 3 International Campus of Excellence “Mare Nostrum”, University of Murcia, 30720 Murcia, Spain; 4 Department of General Didactics and Specific Didactics, University of Alicante, 03690 San Vicente del Raspeig, Alicante, Spain; jose.perez@ua.es; 5 Department of Physical Education, Sport and Human Movement, Autonomous University of Madrid, 28049 Madrid, Spain; aj.munnoz@gmail.com; 6 Department of Evolutionary Psychology and Didactics, University of Alicante, 03690 San Vicente del Raspeig, Alicante, Spain; eliseo.andreu@ua.es

**Keywords:** perfectionism, expectations, self-assessment, physical activity, young people

## Abstract

In the context of physical activity and sport, perfectionism and the regular practice of physical activity are related to psychological well-being and the regulation of psychological resources necessary for adaptation to effort and satisfaction. At the same time, the most active students are also those who show greater appetites for physical education classes. The goal of this work was to identify the influence of perfectionist beliefs and the regularity of the practice of physical exercise on psychological well-being. The participants were adolescents (*n* = 436) aged between 14 and 19 years (M = 16.80, SD = 0.77). They were administered the Multidimensional Perfection Scale, the Psychological Wellbeing Scale, the Global Physical Activity Questionnaire (GPAQv2), and a sociodemographic questionnaire. The results showed, under a non-random and transversal design, that the participants gave important value to physical exercise because they feel both active and vigorous. Regarding perfectionism, the functional aspects of perfectionism (expectations of achievement and organization) correlated positively, while the dysfunctional aspects (fear of committing errors and external expectations) did so negatively with the importance given to physical exercise performed by adolescents; this in turn positively predicted psychological well-being. In this way, the hypothesized model contemplated the relevance of the included variables and reflected the mediation of the degree of importance given to the practice of physical exercise on perfectionist beliefs and psychological well-being. Currently, most physical activity practice proposals for adolescents focus on federated and structured environments for competition, and those that deal with recreational and health-oriented sports are far less common. Hence, “the perfect way of doing sports” for a teenager should be accompanied by cognitive schemes aimed at strengthening psychological resources that allow the regulation of beliefs, attitudes, and behaviors.

## 1. Introduction

The adolescent stage is characterized as being a vital moment of conscious self-discovery, where young people reflect on their own thoughts, maintain their first autonomous experiences with environments that surround and influence them, and where they coexist constantly between the idealization and adaptation of the social norms that surround them [1]. From a functional point of view, adolescents build beliefs associated with their moral, intellectual, and social development, which are translated into formal thoughts that are the bases of their expectations, desires, and aspirations, but also of their frustrations, fears, and conflicts for adult life.

Within such realities, we must highlight the body image [2], sentimental relationships [3], mental health [4], and gender identity [5], together with the relationship between the practice of physical or sport activity and physical and psychological health [6]. Adolescents develop a stable identity, based on beliefs, fruit of both the autonomy with which they learn [7] and their brain development through decision-making [8]. This identity is strongly intertwined with psychosocial functioning and well-being.

Hence, the growing importance of how the adolescent’s interaction with their knowledge and experiences in the practice of physical exercise, and their ideas about well-being [9], suppose elements that guide their responses toward positive or negative evaluations of the same [10,11]. 

Within such a belief system, psychological well-being is concept centered on the development of capacities that empower the adolescent (eudaimonic) and where the coherent and regular practice of physical exercise allows the improvement of resources, such as self-confidence [12,13], mental strength [14,15], self-control and self-regulation [16,17], interpersonal skills [18,19], and emotional adjustment [20,21], thereby constituting an ideal context for people who practice sports which is balanced in such young people [22,23].

The lifestyles and thoughts that establish a hedonistic culture (with constant messages in favor of the immediate satisfaction of impulses and desires) do not allow such an equilibrium in terms of well-being to consolidate, which is influenced by maturational stability (personal and social) [24]. Therefore, the relevance on the "perfect way to achieve things" becomes a factor to be considered in the study of how adolescents act in the face of physical exercise, its establishment as a habit, and its personal value [25,26,27].

As a specific feature referring to concrete actions and behaviors dominated toward the goal [28], the study of perfectionism has allowed it to be contemplated as a transdiagnostic feature regarding how expectations and self-evaluations constitute the basis of such functioning, providing adaptive capacities through the functionality of aspects that can be mastered [11]. In this sense, the benefits that physical activity has on psychological, sociocultural, and cognitive well-being functionalize a large number of mental procedures that allow the modification of behaviors and attitudes that people have toward physical exercise [29].

Despite perfectionism being a personal disposition characterized by striving for flawlessness, it has been associated with positive processes and outcomes in the practice of physical activity (e.g., sport-specific engagement) [30] in both transversal [31] and longitudinal studies [11].

Perfectionism, initially with dysfunctional connotations [32], is seen as the tendency to set too high and unrealistic goals, adhere rigidly to them, and value oneself in terms of achieving them [27,33,34]. In this way, concepts related to mental capacities of effort and emotional stability appear, which could derive from both functional (e.g., perseverance) and dysfunctional (e.g., obsession) patterns of thought, called perfectionist efforts, and refer to the search for self-imposed goals and standards accompanied by harsh self-criticism. On the other hand, mental patterns defined by the pursuit of demanding standards imposed by and for others, perceived external negative evaluation, and the discrepancy between expectations and performance comprise the so-called perfectionist concerns [35,36].

At present, this functional–dysfunctional approach to perfectionism suggests that perfectionism has positive, dynamic, or adaptive aspects, while it also has negative, limiting, or maladaptive aspects [31,37]. Studies reflecting gender in the relationship of practice of physical activity and personality traits determined that girls who were more extraverted and more open to experiences [32,38] also showed more perfectionism and stress perception while practicing exercise, compromising their intentions to exercise [10]. On the contrary, boys were more impulsive, more mentally, structured and sought higher standards (ambitious).

High levels of vitality [39,40], adaptive perfectionism [9,41], and maladaptive perfectionism [42] are associated with vigorous PA. Based on the cognitive and behavioral response when we move toward a proposed goal, perfectionist patterns appear to define the importance and demand of these goals (personal standards), how we are going to achieve them (in a planned vs. disordered path), and what resources we are going to rely on to reach them (strengths–efforts vs. weaknesses–concerns) [43]. Hence, maintaining high levels of exercise involves perfectionist efforts to achieve either by reaching those standards or by not reaching them, as well as by not losing them if they are within reach [44].

The maintenance of persevering effort from the intention of practice [45], a sense of coherence (correspondence with the reasons or goals to be achieved) [46], and the degree of importance given to it [47], function as moderators and predictors of the perceptions of health and subjective well-being for those who perform physical exercise. Likewise, physical exercise practiced on a regular basis in adolescence is related to a healthy lifestyle maintained in time and intensity so that its effects are visible and vigorous, thus strengthening self-esteem [48], the favorable body image [49], and the positive attitude toward psychological changes [50]; aspects that are essential in the face of this functional balance toward adult life in the day-to-day of adolescent life as they grow into adults [51,52].

**Figure 1:**
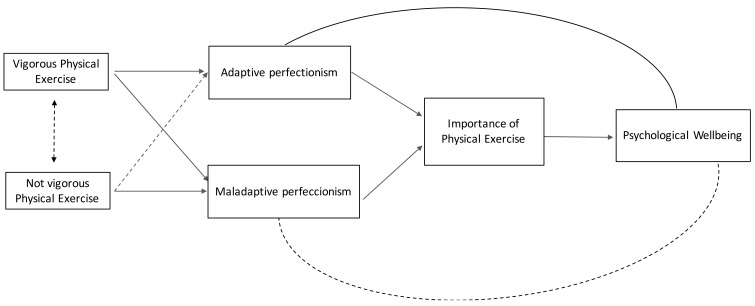
Hypothesized model on the practice of physical exercise, perfectionist patterns, and psychological well-being in adolescents.

The present methodological proposal intends, with a non-random, descriptive, and inferential design, to establish relationships between physical exercise and psychological well-being, with the aim of finding differential and causal explanations, while taking into account perfectionist patterns in a sample of Spanish adolescents. By trying to improve the functional–dysfunctional explanation of perfectionism [33], the hypothetical model (Figure 1) proposes that an increase in the intensity of physical exercise is related to better indicators of psychological well-being, posing the hypotheses that: (a) those adolescents with greater adaptive perfectionism will indicate greater psychological well-being, while those with higher levels of psychological well-being will show less maladaptive perfectionism; (b) adolescents who engage in vigorous physical exercise will show both adaptive perfectionism (functional patterns are activated) and maladaptive perfectionism (activation of dysfunctional patterns); (c) non-vigorous practice will be positively related to maladaptive perfectionism and negatively related to adaptive perfectionism; and (d) a greater importance of physical exercise will be a positive predictor of psychological well-being in adolescents.

## 2. Materials and Methods

### 2.1. Participants 

The sample consisted of 436 Spanish adolescents who performed physical exercise in different municipal sports facilities (*n* = 123; 28.21%) and sports clubs (*n* = 313; 71.79%) from several provinces in Spain (Granada, Murcia, and Madrid). The average age of the participants was 16.80 years (SD = 0.77). The distribution described a composition of 67.43% boys (*n* = 294) and 32.56% girls (*n* = 142). The frequency of practice of physical exercise per week was 3.93 days (SD = 1.24) on average. Among the reasons for exercise given by participants (Table 1), participants emphasized that "being active" was the most important, while “improving your mood” was the least important. In addition, with respect to the attitudes of the participants in the practice of physical exercise, the results indicated that the value given to the exercise they performed was 3.41 (SD = 0.83), that their acquired commitment (0–6) with the practice of physical exercise was 3.04 (SD = 0.51), and that their ability to overcome challenges (0–6) during the practice of physical exercise was 2.97 (SD = 0.80).

### 2.2. Measurement Instruments

*Sociodemographic questionnaire.* Facing the description of the sample, the previous questions considered the age, gender, and number of days that a participant practiced physical exercise. In addition, we asked participants to indicate the main reasons for engaging in physical activity (Table 1). Each question was preceded by the question: “How important is the physical exercise for you…” and each response orientation was distributed on a Likert scale from 0 (nothing) to 6 (a lot). Three items sought to investigate the attitudes of adolescents toward the practice of physical exercise (value contributed by physical exercise, efforts to comply with physical exercise and their ability to overcome), through a Likert scale of 1 (nothing) up to 6 (a lot). 

*Perfectionism.* To measure perfectionism, the *Multidimensional Perfectionism Scale*, which was adapted and validated by Carrasco, Belloch, and Perpiñá [53], was applied to the Spanish population from the original scale *Frost Multidimensional Perfectionism Scale* (FMPS) [54]. The scale was composed of 35 items that came together in four factors of the first order (Expectations of Achievement, Organization, Fear of Errors, and External Influences), two of the second order (Adaptive Perfectionism (Expectations of Achievement, Organization) (e.g., “I am very good at concentrating my efforts to reach a goal”) (and Maladaptive Perfectionism (Fear of Errors and External Influences) (e.g., “If I fail at work, at school or at home, I am a failure as a person”)) and one of the third order (Global Perfectionism). The answer distribution was composed of a Likert scale of five points from 1 (in total disagreement) to 5 (completely agree). In the present study, a second-order structure (bidimensional) was contemplated, obtaining a consistent reliability for the sample with a Cronbach’s alpha of 0.84 for adaptive perfectionism and 0.93 for maladaptive perfectionism.

*Psychological well-being.* The measurement of the eudaimonic psychological well-being [55] centered its attention on the development of capacities and personal growth, which were conceived as the main indicators of the positive operation. For this, the *Psychological Wellbeing Scale* (EBP) was adapted and validated by Díaz et al. [56] in a Spanish population from the original *Scale of Psychological Well-Being* (SPWB) [57]. This scale was composed of 29 items, which came together in six first-order dimensions aimed at offering a complete view of subjective well-being: self-acceptance (e.g., “when I review the history of my life, I am happy with how things have turned out”), positive relationships (e.g., “I often feel lonely because I have few close friends with whom to share my concerns”), autonomy (“I tend to worry about what other people think of me”), domain of the environment (“I find it difficult to direct my life towards a path that satisfies me”), personal growth (“in general, I feel that I’m still learning more about myself”), and purpose in life (“I enjoy making plans for the future and working to make them come true”). In addition, the instrument allowed a second-order structure, composed of a single global factor of psychological well-being. The answer distribution was composed of a Likert scale of six points from 1 (totally disagree) to 6 (totally agree). This instrument showed good reliability, with an internal Cronbach’s alpha consistency of 0.75 for the aforementioned factor.

*Physical activity practice*. To verify the aspects associated with the practice of physical exercise in the sample of adolescents and to measure physical activity, version 2 was administered in Spanish of the *Global Physical Activity Questionnaire* (GPAQv2) [58]. No changes were made to the original content or the text of the questionnaire. The questionnaire consisted of 16 questions regarding physical activity performed in a typical week. These questions gave information on intensity (moderate or vigorous), frequency (days in a typical week), and duration (hours and minutes on a typical day). In the present study, the intensity indicators were considered. The GPAQv2 presented good reliability for this study, showing a Cronbach’s alpha of 0.82.

### 2.3. Procedure

After the approval of the study by the Ethics Committee of Human Research (ID: 1494/2017) from the University of Murcia (Spain), data were collected from different places by access (municipal sports facilities and sports clubs) and closeness (athletes were asked to contact other athletes). In addition, some participants with similar characteristics learned about the study and participated voluntarily. Prior to the administration of the questionnaires to the participants, and in accordance with the ethical guidelines of the American Psychological Association (APA) [59], participants were presented with an informed consent [60] for ethical compliance and data protection, ensuring the rigorousness of the investigation and the privacy of the information obtained. The consent obtained from the parents of all participants was both written and informed. Prior to the start of data collection, the researcher explained the questionnaire and was present throughout its execution to resolve any questions that may arise. A member of the research team was always present to explain, answer questions about the answers, and maintain rigorousness regarding the correct application of instruments.

### 2.4. Data Analysis 

Initially, a descriptive analysis of the sample was carried out through measures of central tendency (means, standard deviations, distribution, and dispersion), discriminant analysis, and partial correlation analysis, thereby controlling the intensity of the physical activity in the two treatments. All analyses were conducted using IBM SPSS version 22 program. Subsequently, the factorial structure of all the measures was determined using a factorial confirmation analysis (CFA), and a structural equation model (SEM) was carried out using the IBM AMOS program (version 19) [61]. The aim was to verify the hypothetical model between sports orientation and psychological well-being, taking into account the mediating value of perfectionism in a sample of Spanish adolescents, who were segmented according to their sporting orientation, i.e., federated or recreational (Figure 1). The estimation method for the SEM was the maximum likelihood along with the bootstrapping procedure to obtain confidence limits for the indirect effects (mid), considering them to be robust estimators [62]. The estimated adjustment indices were: χ^2^/df, the Comparative Fit Index (CFI), the Incremental Fit Index (IFI), the Non-Normed Fit Index (NNFI), the Root MeanSquare Error of Approximation (RMSEA) and confidence interval (CI) at 90%, Standardized Root Mean Square Residual (SRMR), and showing the model’s closeness of fit.

## 3. Results

The intensity of the practice of physical exercise, described as non-vigorous (low or moderate levels, according to the World Health Organization, (WHO)) or vigorous, described discriminant analysis (Table 2), where those adolescents who reported maintaining vigorous activity positively categorized the overall value (<0.00), commitment (<0.02), effort (<0.00), and importance (<0.01) that they gave to the physical exercise they performed. In the same way, it was observed that adolescents who maintained more vigorous physical exercise indicated having more maladaptive perfectionist beliefs (greater external influences and fears of making mistakes) (<0.03) together with a lower psychological well-being (<0.00). On the other hand, no differences were found in those who indicated a more adaptive perfectionism (more organized and with adequate personal standards).

The linear relationships between the variables studied (Table 3) showed causal tendencies while controlling for the intensity of the practice of physical exercise, while appreciating positive relationships between the importance given to physical exercise with perfectionism (both maladaptive and adaptive) and psychological well-being; these relationships were positive between adaptive perfectionism and psychological well-being, and negative between maladaptive perfectionism and psychological well-being.

### Hypothesized Model

The results of the CFA revealed that this study had an acceptable fit of the model and that all standardized factor loads for each item were greater than 0.40, (*p* < 0.01), which also indicated that no item should be eliminated (see Table 4).

**Figure 2:**
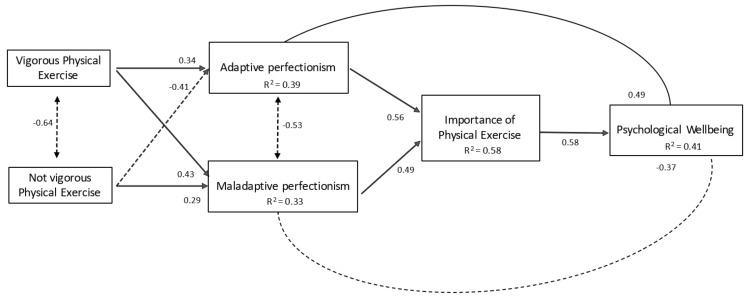
Hypothesized model regarding the practice of physical exercise, perfectionist patterns, and psychological well-being in adolescents.

The adjustment of the tested model (Figure 2) and regression weights contributed to the prediction obtained through bootstrapping. As can be seen, the vigorous practice of physical exercise positively predicted both adaptive (personal standards and organization) and maladaptive (fear of errors and external influences) perfectionism, while the non-vigorous practice of physical exercise (moderate and low levels according to the WHO) negatively predicted adaptive perfectionism and positively predicted maladaptive perfectionism. In turn, both adaptive and disadaptive perfectionism predicted a positive assessment of physical exercise. In the final part of the model, the importance that adolescents gave to the physical exercise they performed positively predicted psychological well-being.

In another order, vigorous physical exercise positively predicted psychological well-being through the mediating value of being more organized and maintaining their stable standards and of giving importance to the physical exercise they performed (β = 0.58; *p* < 0.01). Finally, non-vigorous physical exercise negatively predicted psychological well-being through the mediating value of showing more fear of making mistakes, greater external influences (and therefore greater pressures), and the value of physical exercise (β = −0.26; *p* < 0.00).

## 4. Discussion

The present study attempted to establish relationships between physical exercise and psychological well-being, with the objective of finding differential and causal explanations, taking into account the perfectionist patterns of a sample of Spanish adolescents and the degree of importance they assign to the physical exercise they perform. In addition, the interests and attitudes that the adolescents showed toward physical exercise were initially established, with the purpose of understanding the value of them and their understanding for the indicators of well-being.

In the description of the reasons and interests in the relationships of adolescents with the practice of physical exercise, it was sought to point out different aspects associated with the degree of importance for those who pursue them. Of these, being strong and vigorous or feeling active were the most indicated, reaffirming studies that point to adolescence as the stage where motor competence is consolidated, which is associated with the improvement of healthy aspects, such as weight, cardiorespiratory capacity, and strength [41,63]. Such a description contrasts and complements other studies that previously indicated other types of motives, such as those made by Moreno-Murcia, Cervelló, Huéscar, and Llamas [64] that pointed to fun, being with friends, and a enjoying practice, or that of Gómez-López, Ruiz-Juan, García-Montes, Granero-Gallegos, and Piéron [65], who showed that there was no single reason to maintain active behavior due to the motivations being of an intrinsic nature, such as pleasure, health, and, most importantly, evasion. Rhodes and Kates [66] pointed out, after a systematic review, the relevance of affective involvement as the main source of motivation, while others such as Goguen-Carpenter et al. [67] and Jakobsen and Evjen [68] highlighted the interest, enjoyment, and feeling of competence as the most important elements for the practice of physical exercise in childhood and adolescence.

Taking into account the value that the sample of adolescents attributed to the intensity of the practice of physical exercise, it was considered appropriate to establish different differential analyses regarding the behavior of the variables vigorous practice and non-vigorous practice, showing greater indicators of perfectionism (both adaptive as maladaptive), psychological well-being, and the degree of importance of the physical exercise they performed [69,70,71,72]. Such results reflected both the ambiguity of the perfectionist patterns in the perception of physical exercise and the relevance of vigorous activity as a source of well-being (also bearing in mind that this was one of the reasons most noted by the participants).

Although perfectionism maintains a multidimensional nature, the existence of ambivalent patterns is common in its relations with sports practice under constant functionality and dynamism [11,28,31,32,73]. Both the functional orientation (which allows one to adapt more effectively and with greater awareness) and the dysfunctional orientation (which orientates the reactivity toward fear and gives greater value to external pressures) feed off a greater intensity in the practice of physical exercise [74,75]; the first reaffirms such beliefs of security and focus (e.g., desire, commitment, or audacity), and the second avoids the associated negative symptomatology (e.g., greater anxiety, fear, or low self-esteem) and focuses toward the adolescents’ health [27,32,34].

As expected, as previous studies on physical exercise and psychological well-being in adolescents have pointed out [6,40], self-evaluation regarding how important physical exercise is performed is an adequate predictor of well-being in adolescence [6,9]. Precisely, the relevance observed in the present study indicated that the importance attributed to physical exercise was different when it came from the maladaptive perfectionist pattern, although later it was configured to be a positive mediating value toward psychological well-being [11,63]. 

Teenagers who practiced a little vigorous exercise pointed to a greater inappropriate perfectionism, however patterns based on concerns, doubts, and fears activated the intention to practice physical exercise. With a focus on health conditions, fears and concerns could be established as adequate regulators to generate intentions that promote healthy attitudes [13,14,15,23]. 

These data offer interesting and new ways regarding which type of beliefs have a greater influence on the assessment process of a task, and how this assessment induces changes toward positive and negative tendencies between the relationship of maladaptive perfectionism and well-being [17,22,30]. Other previous works offered similar results, associating psychological well-being with self-oriented perfectionism (to grow on the own perfectionist beliefs), behavioral imbalances, and psychological discomfort alongside prescribed social perfectionism (to grow from what others dictate) [21,24,39,43,45,52].

## 5. Conclusions

Adolescence has been identified as the stage of the life cycle where essential aspects of the character are configured from identification with others. In this stage, functional or dysfunctional resources begin to be discovered, before the more autonomous awakening of psychosocial aspects that determine adult life (e.g., achievement expectations, personal goals, social relationship skills, help behaviors, impulse regulation); dimensions that regulate and motivate the behavior of the individual in the future, and which maintain a direct relationship with an adolescent’s perception of well-being.

The practice of physical activity on a regular basis in school and outside of school is established as both a behavior and a regulating habit of these learning individuals, where a young person with active habits puts into practice such resources through their physical efforts, stimulating basic systems of human functioning (e.g., cerebral, hormonal, social, psychological, identity) and influencing their perceptions about themselves and their surroundings. In this way, they build functional patterns that facilitate individualization of what they deem perfect to achieve objectives, or at least learn to manage different personal strategies that allow them to value their proximity to their goals.

After observing and analyzing the results obtained with detail and rigorous scientific procedure, it is evident that the knowledge of these indicators is important when managing any process of psychological change aimed at improving sports skills. With proper planning and ordering of the different levels of physical exercise, psychological well-being will be affected. The regular practice of physical exercise with considerable intensity (within a balance in terms of effort, intention, and realization) achieves physical improvement and allows the development of more positive perceptions of one’s own image and, as a consequence, of elements such as self-esteem [50] and well-being. Thus, a positive assessment of physical exercise predicted good indicators of psychological well-being in adolescents who participated, although it would be of great importance to consider whether the assessment of the importance maintained in the long-term (intensity and duration), would generate the same linkage as a transversal study.

This study presents a series of limitations focused on the difficulty of obtaining the permits for data collection; a process that required a considerable temporary cost. In addition, the use of different researchers required a brief preparation session to agree on the application protocol. Accepting that the transversal methodology allows fewer options for generalization when the samples are not very large, the results obtained provide relevant information that will have to be corroborated in future empirical proposals. In spite of this, the obtained data allowed the confirmation of our hypotheses, as well as the obtainment of a design that fit the purposes of this investigation. 

Future proposals that arise from the data obtained involve raising concerns about the way to connect adolescents with physical exercise, accepting some individual differentiation in terms of their mental patterns, cognitive styles, and psychosocial responses to physical exertion. In addition, we will consider studies to be suitable that allow adolescents to conceive attitudes to remain active; that is to say, studies based on the conviction of the utility that the practice of physical exercise contributes to well-being and the healthy physical conditions, instead of orienting such experiences of physical activity toward less stable aspects, such as competitiveness or rehabilitation. 

Different personal variables (such as self-concept, impulsiveness, search for sensation, mental toughness, etc.) must be considered in future work, as well as resources and psychological skills (such as resilience, self-regulation, or skills with others). In this way, we can know the multidimensional nature of how perfectionism built individually and socially intervenes in the learning of the psychosocial response in adolescents.

At the same time, the establishment of designs that allow contextual differentiation will allow them to be observed under aspects such as gender, social contexts, profiles of teachers, coaches, parents, etc., thus allowing generalization regarding the explanation of established predictive relationships or establishing different contexts for teaching styles or social support in sports practice.

The definition of “perfect and unique paths” to experience well-being should not be the goal of scientific advances; rather the opposite, since this would be exclusive and reductionist. On the contrary, the integration of different strategies and the relationships between variables, from multi-level and multi-causal points of view, will allow interactions of adolescents with the contexts of physical exercise to serve for the functionality of their resources toward an active life and, ultimately, the ability to adapt to understand and interpret their states of effort and welfare.

The WHO has been pointing out for decades that an adequate balance regarding the practice of regular and daily physical exercise allows both physical and psychological benefits. To engage in sport requires a balance between approaching the desired objectives in an ordered and functional way (attitudes which will help to reach objectives) or agonic, doubtful, and dysfunctional (which can cause imbalances when reaching objectives). This will have repercussions on the intention to continue with the mentioned practice, and on the resulting psychological well-being. Athletes and coaches, since adolescence, should have clear processes for the approach and learning of the advances that are generated with the practice of sport. Starting from the structural aspects of teaching and accessibility to sports practice (e.g., competitive vs. recreational orientation and teaching vs. technification), will help to develop perfectionist patterns that allow functionality in the practice of physical exercise, which will in turn influence the intention of practice based on trust, while dysfunctional patterns will be based on a path of doubts, fears, and worries.

## Figures and Tables

**Table 1:** Descriptive statistics and variance of reasons for the practice of physical exercise.

*n* = 436 Age = 16.80 Years Old (SD = 2.93) “I Practice Physical Exercise for…”:	Range	M(SD)	Kurtosis	Asymmetry
To reduce weight	0–6	2.74 (1.84)	1.32	0.26
To feel active	0–6	4.03 (0.92)	1.43	1.74
To improve quality of life	0–6	2.95 (1.93)	0.36	0.07
To be strong and vigorous	0–6	3.71 (1.64)	1.04	1.30
To improve mood	0–6	2.38 (1.22)	0.83	1.33

**Table 2:** Discriminant analysis according to the intensity of PA, perfectionism, and psychological well-being in a sample of adolescents.

Variables	Vigorous (*n* = 193) M(SD)	Non Vigorous (*n* = 243) M(SD)	(*λ*)	F	CE (Sig.)
**Importance of Physical Exercise**					
Value of the PE in general	4.41 (1.32)	2.89 (1.74)	0.74	7.26	5.29 ^**^
Commitment to the PE	4.63 (1.46)	2.57 (1.23)	0.62	3.81	3.53 ^*^
Effort for the PE	3.94 (1.05)	2.37 (1.34)	0.81	8.93	4.90 ^**^
Importance of the PE	4.32 (1.27)	2.61 (1.44)	0.69	6.04	4.68 ^*^
**Perfectionism**					
Adaptive perfectionism	3.26 (1.38)	3.13 (1.07)			
Maladaptive perfectionism	3.82 (0.93)	2.76 (1.04)	0.92	8.53	4.34 ^*^
**Psychological Well-Being**					
Psychological Well-being	4.39 (1.35)	2.86 (0.67)	0.87	6.95	8.03 ^**^

*n* = 436. PE: physical exercise. Vigorous: ≥3 days per week of PA (minimum 60 physical exercise min/day). Non-vigorous (moderate or low): ≤3 days per week of PE (minimum 60 min/day). *p < 0.05; ** *p* < 0.01. F: Snedecor scores. CE: standardized coefficients.

**Table 3:** Partial correlations between perfectionism and well-being when controlling for the intensity of physical exercise.

Variables	M	SD	Range	1	2	3	4
Importance of physical exercise	3.14	0.76	0–6	-	0.53 ^**^(0.00)	0.48 ^*^(0.01)	0.56 ^**^(0.00)
Adaptive perfectionism	2.94	1.07	1–5		-	0.37 ^**^(0.00)	0.67 ^**^(0.00)
Maladaptive perfectionism	3.02	0.84	1–5			-	−0.49 ^**^(0.00)
Psychological well-being	3.78	1.68	1–6				-

Control variable: intensity of PE practice; *p < 0.05; ** *p* < 0.01.

**Table 4:** Confirmatory factor analyses hypothesized—goodness of fit indices.

Model	χ2	*df*	RMSEA	(90% CI)	SRMR	NNFI	CFI
Model 1	451.35	154	0.028	(0.014–0.032)	0.061	0.925	0.932

For all values of χ^2^, *p* < 0.00; df: degrees of freedom; 90% CI: 90% confidence interval for the RMSEA (Root MeanSquare Error of Approximation); SRMR (Standardized Root Mean Square Residual); CFI (Comparative Fit Index); NNFI (Non-Normed Fit Index).
